# End of life care in sub-Saharan Africa: a systematic review of the qualitative literature

**DOI:** 10.1186/1472-684X-10-6

**Published:** 2011-03-09

**Authors:** Marjolein Gysels, Christopher Pell, Lianne Straus, Robert Pool

**Affiliations:** 1 Barcelona Centre for International Health Research (CRESIB, Hospital Clínic-Universitat de Barcelona), C/Rosselló 132 SA 1ª, Barcelona 08036, Spain; 2 Centre for Global Health and Inequality, University of Amsterdam, Spui 21 1012 WX Amsterdam, The Netherlands

## Abstract

**Background:**

End of life (EoL) care in sub-Saharan Africa still lacks the sound evidence-base needed for the development of effective, appropriate service provision. It is essential to make evidence from all types of research available alongside clinical and health service data, to ensure that EoL care is ethical and culturally appropriate. This article aims to synthesize qualitative research on EoL care in sub-Saharan Africa to inform policy, practice and further research. It seeks to identify areas of existing research; describe findings specifically relevant to the African context; and, identify areas lacking evidence.

**Methods:**

Relevant literature was identified through eight electronic databases: AMED, British Nursing Index & Archive, CINAHL, EMBASE, IBSS, MEDLINE, PsycINFO, and the Social Sciences Citation Index; and hand searches. Inclusion criteria were: published qualitative or mixed-method studies in sub-Saharan Africa, about EoL care. Study quality was assessed using a standard grading scale. Relevant data including findings and practice recommendations were extracted and compared in tabular format.

**Results:**

Of the 407 articles initially identified, 51 were included in the qualitative synthesis. Nineteen came from South Africa and the majority (38) focused on HIV/AIDS. Nine dealt with multiple or unspecified conditions and four were about cancer. Study respondents included health professionals, informal carers, patients, community members and bereaved relatives. Informal carers were typically women, the elderly and children, providing total care in the home, and lacking support from professionals or the extended family. Twenty studies focused on home-based care, describing how programmes function in practice and what is needed to make them effective. Patients and carers were reported to prefer institutional care but this needs to be understood in context. Studies focusing on culture discussed good and bad death, culture-specific approaches to symptoms and illness, and the bereavement process.

**Conclusions:**

The data support or complement the findings from quantitative research. The review prompts a reconsideration of the assumption that in Africa the extended family care for the sick, and that people prefer home-based care. The review identifies areas relevant for a research agenda on socio-cultural issues at the EoL in sub-Saharan Africa.

## Background

End of life (EoL) care is an important public health concern [1,2] predominantly due to the large number of people it affects. In sub-Saharan Africa HIV/AIDS is the most pressing concern with 22.5 million infected people in 2009, which is two thirds (68%) of the global population living with HIV/AIDS [3]. Although anti retroviral therapy (ART) is becoming more accessible, the prevalence of HIV across sub-Saharan Africa may rise [4]. The current disease burden of HIV is augmented by cancer and other non-communicable disease and with an aging population, the incidence of cancer has been predicted to grow substantially [5].

Over the last 30 years, since Africa's first hospice was set up in Zimbabwe [6,7], various models of EoL care have emerged in sub-Saharan Africa [7]. These include home-based care, hospital units, freestanding inpatient hospices, day care and hospital support teams [7]. Nonetheless, due to poverty, lack of resources and infrastructure, care provision at the EoL is scarce across the continent [8]. A study mapping EoL care initiatives showed that there were services in only 26 of 47 countries, and only in Uganda, South Africa, Kenya and Zimbabwe are services reaching a level of integration into the existing health system [9]. Indeed, across the continent the response to the need for EoL care has been piecemeal with governmental and non-governmental organizations playing a range of roles [6].

There is also a paucity of research [1,10]. End of life care in sub-Saharan Africa still lacks an evidence-base which is urgently needed for the development of effective service provision [10]. A systematic review of research on EoL care in sub-Saharan Africa uncovered 'a wealth of clinical and academic experience but a dearth of methodologically robust evidence' [8]. Apart from the lack of service evaluation and outcome data, very little research has addressed the EoL care needs of patients and their carers. Evidence is needed to address deficits in care, improve care and to ensure that palliative care can secure its increasing share from national budgets and allocate these resources in a just way [11].

In light of the pressing awareness of the need to increase coverage of EoL care services on the African continent, emphasis is often placed on the generation and appraisal of evidence that reports on efficacy and effectiveness. Undoubtedly, such evidence is important and measurable outcomes are valuable to the improvement of service quality. Nevertheless, evidence based on the experiences of actors involved at the EoL, and the historical, political, cultural context that influences the way people approach illness and death should not be overlooked. This type of evidence is needed to ensure that interventions developed on the basis of the needs of Western populations are not transplanted into African contexts with their varied epidemiological, cultural and organisational set ups [1].

The systematic review is a recognised tool for the synthesis and evaluation of quantitative health research [12,13]. However, despite documented limitations [14], there has been increased recognition of the role of systematic reviews of qualitative research in developing an evidence base for care [15]. In spite of this, no systematic review of the qualitative research on EoL care in sub-Saharan Africa has been published. A synthesis of research undertaken with qualitative designs will open up an area hitherto consulted less often by palliative care service planners; it will make the findings from diverse sources available to the policy agenda and may lead to better-informed decisions regarding funding of services and future research.

This article aims to synthesise qualitative research EoL care in sub-Saharan Africa to inform policy, practice and further research. It seeks to address the following questions: what are the areas of existing research? Which findings are specifically relevant to African contexts? Which areas are lacking evidence?

## Methods

Relevant literature was identified through searches using nine electronic databases: AMED (1985 - August 2010), British Nursing Index & Archive (1985 - August 2010), CINAHL (1951 - August Week 4 2010), EMBASE (1980 - 2010 Week 34), IBSS (1951 - August 2010), MEDLINE (1950 - 23rd August 2010), PsycINFO, (1967 - August Week 4 2010), the Social Sciences Citation Index (1956 - August 2010) and African Journals Online (all years). In light of the limitations of electronic databases searches for the identification of qualitative research, hand searches of relevant journals and the bibliographies of identified articles were also conducted. See Additional file 1 for further details of the search strategy.

Published primary studies on EoL care in sub-Saharan Africa that used qualitative methods were included. Only published studies were included to ensure a minimum quality of research. Papers using both quantitative and qualitative methods were included if the study was recognised as a mixed method study or if the qualitative methods could be considered as a separate section of the study. This guaranteed that study designs were well-considered. Exclusions were made if the article was not based on research in sub-Saharan Africa, did not relate to EoL care, was not primary research, did not include a separate qualitative component with a clearly defined purpose, was not in English. The study selection criteria were applied by one author in consultation with the at least one other.

**Figure 1:**
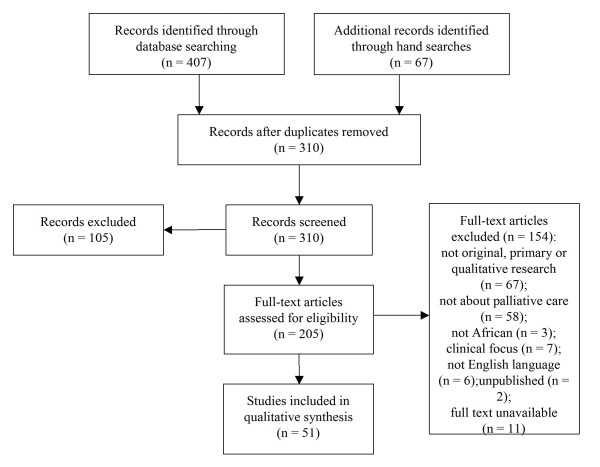
The article selection process

References were managed using Endnote X2. Identified references from all sources were imported and duplicates removed. Titles and abstracts were read, and if the source was suitable for inclusion, the full article was obtained and read. Authors were contacted when the full text version could not be accessed. Figure 1 illustrates the selection process followed. For the articles included in the qualitative synthesis, quality was independently scored by two reviewers with a standard grading scale [16] and differences in scores were resolved through discussion. Ten areas (title and abstract; introduction and aims; method and data; sampling; data analysis; ethics; bias; results; transferability or generalizability; and, implications and usefulness) were awarded grades ranging from one (very poor) to four (good), providing a maximum score of 40.

Information was extracted from the articles and tabulated (Additional file 2). This formed the basis for examining the extent, range and nature of the literature. The next step focused on the synthesis of the findings, whereby themes across multiple studies were developed inductively through constant comparison of findings, and these are represented by the headings in the results. These findings were broadened out by placing them in the context of the wider literature, which provided a view of what is specifically relevant to African contexts, and their significance for the development of EoL care in general.

## Results

### The nature of the studies

Fifty-one articles are included in this review. The majority of the articles focused on HIV/AIDS (38); were based on research in South Africa (19), and were ranked as 'good' (30 studies scored over 30 out of 40 on the standardised grading scale [16]). An important number of studies (20) also focused upon home-based EoL care. Tables 1, 2, 3, and 4 provide a summary of the characteristics of the articles included in the qualitative synthesis.

**Table 1:** Location of data collection for the studies included in the qualitative synthesis

Country (or countries) of data collection	N*
South Africa	19
Kenya	6
Uganda	7
Zambia	2
Tanzania	3
Botswana	6
Malawi	1
Democratic Republic of Congo	1
Ghana	2
Mozambique	1
Namibia	1
Rwanda	2
Togo	1
Lesotho	1

*N totals ≥51 as some studies took place in more than one county

**Table 2:** Participants included in the studies incorporated in the qualitative synthesis

Participants	N*
Caregivers (informal)	27
Health Professionals	24
People requiring palliative care	17
Community members	8
Family post-death	2

*N totals ≥51 as some studies included more than one type of participant

**Table 3:** The quality of the articles included in the qualitative synthesis

Quality of article*	N
Good (30-40 out of 40)	30
Fair (20-30 out of 40)	21
Poor (10-20 out of 40)	0
Very Poor (1-10 out of 40)	0

* Independently scored by two reviewers with a standard grading scale (Additional file 2)

**Table 4:** The illness that articles included in the qualitative synthesis focused on

Illness focused upon	N
HIV/AIDS	38
Multiple or non-specified	9
Cancer	4

### The burden of EoL care

In most of the articles, EoL care was reported to be a significant financial, physical, or social burden for carers. Providing appropriate food, medicines and water financially impoverishes carers and increases the burden of care [17-27]. Funeral costs were also mentioned as a large economic burden [27,28]. Time spent on caring meant less time for food [17] and livelihood production [24], and carers sometimes sacrifice their own resources for the patient [23].

The physical consequences of caring for someone with EoL care needs were mentioned in several studies [18,22,24,25,28-30]: carers were exhausted and developed pains after moving patients and carrying out strenuous household tasks. Several studies also described how HIV positive caregivers' health was compromised through caring for someone else [20,21,25]. The studies likewise provided evidence of the psychological impacts of caring; the emotional demands of caring resulted in loneliness, depression, and isolation [18,23,24,28,30-33]. Factors that increase the stress of caring include the stigma of the patient's illness, parental carers needing to come to terms with their child's HIV status, patients being abusive and unpleasant to carers, and a lack of knowledge regarding the illness or best care practices [17,23,25,30,31]. Grant et al. reported communication difficulties in families due to a fear of discussing care and illness, as this would increase their vulnerability to "evil forces" [19].

The burden of care is also often compounded by gender and age related issues. Many authors discussed the material and emotional burden of care that women bear for their children, orphaned children, and relatives [23,25,26,28,30,31,34-37]. Moreover, Matukula et al. argued that funds to pay for care should be directed specifically to women [21]. In some instances, younger female carers face physical and sexual abuse from family members [28]. Thomas' study however questioned the assumption that the burden of care falls on women [24]. In regard to age, elderly carers in Togo [22] and South Africa [27] faced additional challenges as a consequence of role reversal: elderly parents had to care for their adult children, and to cater for the needs of their grandchildren [27]. The health of older carers can suffer as a result of their care-giving, especially in Uganda where children seek parental care only when they are very sick and require intensive care [28,30]. The burden of care borne by older carers also impacts upon the living arrangements and family relationships [38].

Low levels of support from other community members results in a social burden of EoL care for carers [20,25,26,31]. Identified causes of this lack of social support included caregivers' fears of community members' reactions towards people with HIV/AIDS, and carers withdrawing from social activities due to the demands of caring [17,21,23,25,28,30]. Additionally, the nature of the illness can cause social and personal disruption [24]. Although carers had previously sought help from traditional healers, due to the costs and lack of confidence in their services, this was reported to be becoming less common in Botswana [39].

Several studies demonstrated how in households the care, illnesses, and deaths of family members create a complex burden of care [20,23,28,30,36]. Furthermore, a patient's role within the household may change due to the inability to reciprocate care or financial support and place strain on personal relationships [22,24]. Given its importance in care provision, two studies argued that approaches and policies towards EoL care should be focused at the household level [20,21].

Although the negative impacts of the care burden are commonly reported, Akintola [40] detailed some of the rewards that carers receive in exchange for their assistance. The author argued that although the rewards do not outweigh the burden of care for volunteers caring for people living with AIDS, rewards include improving their own health, enabling them "achieve self-growth", acquiring new skills and gaining recognition from the community.

The burden of care for health professionals providing care for HIV/AIDS patients is also highlighted. Staff in South Africa and Zambia felt emotional stress, had fears of infection, and were unable to cope with the loss of patients [32,41-45]. Their burden of care was worsened by lack of hospital resources, such as beds, staff or medicines [25]. Community carers faced difficulties, such as not being welcomed by patients' families for fear that their presence would stigmatise the family [25] and undertook tasks that were not their responsibility such as arranging funerals [45].

### Training, support needs and tools

The improvement of training and support for informal and professional carers appears as a recurrent recommendation to lessen the burden of care. Identified areas of training include: basic hygiene, clinical aspects of care, symptom management (including fear of opioid addiction), prevention of patient to carer infection, care for children, counselling for patients and families, home-based care, information provision, and psychiatric and bereavement care [17,18,23,25,28,30,33,34,36,42,44-47]. Mtalane et al. recommended training for professional carers regarding the emotional, spiritual and cultural needs of the dying patient [48]. In contrast, Uys (2003) found that professional carers were comfortable meeting patients' spiritual needs [48,49]. Two studies that report the evaluation of an education strategy in rural Uganda [50,51] recognised the core role of education for service development.

Selman et al. [47] identified that the information needs of patients receiving EoL care and their carers regarding symptoms, symptom management and disease cause, progression and treatment were commonly not met. The poor information supplied by healthcare personnel working in EoL care services in South African and Uganda led to high levels of distress amongst patients and their carers. The authors therefore recommended generalist EoL care training for all clinical staff, which should include communication and basic counselling skills. Information provision should also be individually tailored, proactive, open, documented and for both the patient and carer [47].

Caregivers' psychological burden is the focus of identified unmet support needs. Counselling services, respite care, stress management courses and support meetings for carers need to be developed or improved [17,18,28,42-44,49]. Furthermore, EoL care service managers need guidance in the management of HIV positive staff, especially in regard to dealing with workplace stigma [43].

Nurses in Lesotho, who were interviewed about their knowledge of and attitudes towards EoL care and the WHO Integrated Management of Adolescent and Adult Illness (IMAI) guidelines, demonstrated limited knowledge of the presence of the palliative care booklet in the IMAI guidelines [52]. In spite of their lack of familiarity with this tool, the nurses identified the need for "holistic" EoL care for people living with HIV/AIDS.

### Place of care

Eleven papers focused on place of care in advanced illness. Hospice care initiatives are still a recent development and an evaluation of the hospice referral process was reported to promote improved access in South Africa [53]. One study in a cancer palliative care setting addressed the related issue of information behaviour, pointing to its complexity and the need for further investigation [54].

Four studies were undertaken in the context of home-based AIDS projects in South Africa [25,42,45,49], and one study on an intervention providing home care to a population with EoL care needs in Kenya [55]. Several studies targeted home-based care, with family members as the sole providers of care, giving insight into patients' preferences for place of care and the conditions needed to supply home-based care [22,26,33,34,37,39,56-58].

Uys et al. [49] performed a multi-method study in seven sites in South Africa working with the integrated community-based home care model and found a relationship between dying at home and "good death". From which the authors concluded that this is the model of choice for patients with AIDS and asserted that home-based care is not a second best option for developing countries but has the capacity to improve the illness and dying experience. The study highlighted the need for outreach programmes and poverty relief as patients often sought hospital care in order to obtain food rather than treatment.

In Hunter's 2005 study [25], patients and informal carers expressed a need for professional care because they encountered problems accessing clinics. Uys and De Saxe described the practices of community caregivers (CCGs) in a home-based AIDS care project [42,45]: CCGs were positive about their contribution [45], however, CCGs received little salary and had low professional status which led to high levels of staff turnover [42,45]. The degree of community caregiver supervision also had an important effect on the success of the projects.

A mixed method study on the EoL care needs of HIV/AIDS patients in Rwanda found that the second most commonly perceived need after medical, psychosocial and financial care (77%) was home-based care (47%) [57]. A later paper by the same authors in the same study site, focused on terminal AIDS patients' preferred place of care [58]. Whilst the quantitative data showed that 67% of participants preferred hospital care, the qualitative findings attributed this to the lack of home care provision in Rwanda and to patients having lost family members in the 1994 genocide war who would have otherwise taken care of them.

Four studies [22,33,34,56] found that home care is not the evident choice at the EoL. Olenja's study of community attitudes towards home care for patients with AIDS in Kenya illustrated how home care was seen as unrealistic in a context of poverty, stigma and lack of knowledge of how to care for a person with AIDS [34]. Two studies [22,33] from different regions reported similar findings that represent the carers' view: once a patient was discharged from hospital, carers found themselves unsupported and on their own. The capacity for care, traditionally available through the extended family structure, had changed permanently due to AIDS. Only the elderly directly related to the patient felt responsible to provide care for their children.

Murray et al. [56] provided insight into how palliative care patients in Kenya experienced their stay in the hospital: patients felt alone and removed from their family, who were not always able to visit them due to the cost of transport.

### Good death versus bad death and stigma

Two ethnographic studies focused on conceptions of death in Ghana [59,60]. In the 2002 study, Van der Geest explored older people's views about death in a rural town in Ghana [59]. There was no fear of death. Rather, people spoke of death as a welcome visitor that brings peace and rest. Respondents maintained an agnostic stance towards the future. In Van der Geest's later study [60], the accounts of the deceased relatives' last moments were not dominated by medical interventions and bad deaths were deaths that come too early, deaths that were believed to be punishments for sins. Those who died a bad death did not receive a proper funeral; a good funeral is part of a good death.

In rural Tanzania, using ethnographic methods Dilger [29] identified a moral discourse around HIV/AIDS; understood as a bad disease, HIV/AIDS was brought by indecent behaviour of family members who migrated to the cities and this legitimised the poor quality care that in some cases people received. Moreover, HIV-infected migrants often returned to their rural families at the final stage of illness, placing increased burdens on their relatives.

Studies from Kenya and South Africa also revealed that the stigma surrounding HIV/AIDS was due to its association with immoral behaviour [35,61]. Another study from South Africa explained the stigma in terms of the 'terminality' of HIV/AIDS [62]. People were aware of biomedical explanations for its cause but contested them with views based in local cosmologies and belief systems [29,35,61]. In some instances, but not all [38], the stigma of HIV/AIDS impacted on both the patient and the caregiver. In some cases stigma led to problems accessing local services, rejection and the breakdown of social support [20,22,24,28]. And in Botswana, there was a 'culture of silence', due to the stigma and denial of AIDS [33].

In two studies from Kenya and Tanzania, death from HIV/AIDS was described as particularly disturbing because it supposed a 'permanent death' [29,61]: those who died from AIDS were denied the status of ancestors, and this threatened the continuity of the clan and community. Families attached great importance to burying the deceased in their village of origin, due to ancestral attachment of the deceased to their home. However, the shame linked to AIDS disrupted this cultural practice, leading to private funerals. The prospect of such a burial caused anxiety and affected people's dignity, impacting on their relatives and subsequent bereavement.

### Bereavement

Three studies by Demmer [31,32,36] addressed AIDS-related loss in South Africa. One [31] study highlighted how grief was complicated due to the silence around HIV/AIDS and carers only realised patients' status in the advanced stages of illness. Demmer's 2007 article on the impact of loss on people's daily lives found that, because of poverty, grief was a luxury and concerns around survival took priority [36]. Another study examined how professional carers helped their clients deal with AIDS-related loss and grief and how it affected them personally [32].

### Culture-specific experiences and approaches to symptoms, illness, death and caring

Two studies addressed the culture-specific responses to symptoms and illness and the implications for its management [46,63]. Bor [63] compared experiences of patients suffering from Kaposi sarcoma in the UK, USA, Zambia and Botswana, and found that the setting and culture mediate social responses to Kaposi sarcoma and disfigurement. Beck's ethnographic study in South Africa [46] identified cultural variability of cancer as an illness, pain expectations, tolerance and expression, and treatment practices. Barriers to effective pain management were caused by the lack of standards in practice, knowledge and resources. Relations between patients and health workers and problems with communication extended to pain assessment and management [46].

By focusing on how professionals think about and practice disclosure of a terminal cancer diagnosis in an African setting, Harris et al. [64] showed the importance of cross-cultural differences in medical practice. Their study was conducted in one of the largest academic hospitals in Africa and could therefore contrast the views of both Tanzanian and expatriate physicians. Although Tanzanian physicians had been exposed to medical practices in foreign settings and were familiar with the values of Anglo-American bioethics they opted for a counselling approach derived from culture-specific discursive practices.

## Discussion

### The focus of the evidence

Nearly 40% of articles in this review are based on research from South Africa. Many countries in Africa are not represented at all and others are under-represented. This highlights the lack of research on socio-cultural aspects of EoL care in African countries outside South Africa.

There is a spread of participants across the studies: patients, informal carers, or professional carers. Although this provides a range of perspectives, few studies involve community members. Incorporating voices from the wider community would enable EoL care to be situated within the local social and cultural context. Many papers in this review focused on HIV/AIDS whilst other illnesses were under-represented. Although these studies cannot address all the issues that arise in other life-limiting conditions, they can serve as a basis for the development of EoL care across contexts specific to sub-Saharan Africa.

### Caring for patients with EoL care needs

The qualitative studies provide ample evidence on the experience of informal carers, which is an area that tends to be under-researched [65,66]. Carers appear most often as sole carers providing the totality of care, without respite. In the EoL care discourse on carers in other parts of the world, they are seen as in a double position, as part of the caring team, as well as being in need of care themselves [67]. In the reviewed studies that report carers' experiences, they lacked support, and there was no attention to carers' needs.

The studies show that mostly women take on the role of caring, without expertise, and on top of other domestic, income-generating and childcare responsibilities. Due to the complexity of relationships and duties, the studies recommended that care needs be assessed from a household perspective, which is in line with the EoL care approach that treats the family as the 'unit of care' [68]. The findings refute one of the myths about the African caring system, that of the inexhaustible capacity of the extended family to withstand crisis [69]. HIV/AIDS has eroded this system of mutual obligation by affecting several family members at once, changing provider and dependency relations in unexpected ways. The reviewed studies demonstrate that the responsibility for informal care had in many cases shifted to the elderly and children [22,23,30].

Health professionals, both in a hospital [44], as in a home-care context [42], find their work challenging due to heavy workloads in under-resourced and risky conditions, for which they received low wages. This leads to burn-out and attrition of expertise among those providing the hands-on care. Training provided for health professionals should take into consideration these challenges and prepare them appropriately.

In spite of the burden of care that carers experience and their huge unmet support needs, positive aspects of caring emerge from the reviewed studies [40,42,44]. Care is provided out of love or empathy in many cases and can bring fulfilment and self-respect. Understanding the challenges as well as the rewards and functions of caring and how crises are overcome is essential for building appropriate support systems for carers reducing their burden of care, and, in the case of volunteer carers, reducing attrition rates [40].

### Place of care

The HIV/AIDS epidemic has stretched public health services in sub-Saharan Africa beyond their limits. Therefore the principal responsibility for providing care for people with HIV/AIDS has been taken up by families and communities. In response to this need, community/home-based care has become a central concept. Home-based care developed according to local contexts and realities and within the limits of scarce resources [18,70]. Influential publications, such as the WHO Technical Report on home-based care, underscore the view that primary health systems in developing countries can form the basis for sustainable, cost-effective, long-term care [71].

The studies report how these programmes function in practice and what is needed to make them effective. Apart from the successes, such as that identified by Uys [45], various challenges were found. For example, the ethnographic approach of Hunter's study provided a critical view on home and community-based care. The author's analysis of policy approaches demonstrated the adverse effect of promoting home-based care without the health system dedicating the necessary resources to home-based care, resulting in 'home-based neglect' [25].

From the studies, preference for place of care appears as complex as in Europe [72]: depending on family circumstances, available facilities, illness stage, ideal versus real preference, and the perspectives of those involved. The studies reveal a preference for institutional care, which contrasts with arguments for home-based care and previous survey results [73,74]. The evidence on preference for hospital care came from patients and health professionals in the Rwandan post-genocide context [58]. It also came from informal carers confronted with the unsustainable burden of caring single-handedly for a dying relative [22,33] and from a community confronting stigma [34]. Only one study documented patients' experience of longing for home-based care in a hospital setting [56]. More evidence is needed on preferences for place of care and its determinants.

### The influence of culture on illness, death and bereavement

Pain management has clear cultural dimensions. Although the effectiveness of the WHO step-wise pharmacological approach to pain relief has been shown, barriers to appropriate implementation remain. The WHO model recommends a strategy of government policy, education and drug availability, however, it does not address the cultural beliefs that influence expectations and treatment decisions about pain in specific contexts [46].

This review shows that studies on cultural issues at the EoL in an African setting are scarce. Those based on longer-term ethnographic work were most informative: the studies from Ghana [59,60] gave insight into the experiences and meanings ascribed to death and dying by the very old. Differences, as well as universals, in conceptions of death and dying need further investigation to assess their use in interventions or clinical assessment tools.

An emphasis on the burial, rather than the dying person was noted in Ghana [59,60]. The sickbed and dying tend to be confined to the seclusion of the house, while the funeral is public. The private/public distinction and associated moral value of spending scarce resources on uncertain outcomes may determine the contrast between pre- and post-mortem care and attitudes to caring. To what extent these norms prevail, and under which conditions this occurs, needs to be studied in order to inform clinical practice, as this can lead to a loss of hope at the EoL.

Three studies from eastern and southern Africa on HIV/AIDS provided evidence of the detrimental consequences of understanding HIV/AIDS as an immoral illness [29,35,61]. Long-established values and traditions, which give meaning and purpose in life, and death were affected by this understanding. The studies' findings show the significance of meaningful rituals: funerals traditionally fulfil a key role of restoring the social order and are important public events.

Stigma [75] is a cross-cutting theme emerging from the studies; it was discussed mainly in the context of HIV/AIDS, but also in regard to other terminal illnesses. It is important to include secondary stigma, the discrimination of carers due to their association with the ill, in future attempts to reduce stigma [44]. Interventions to reduce stigma need to go beyond the individual and psychological, and ethnographic information needs to be collected to enable culturally sensitive interventions to be carried out [76].

The review identified only three studies on the bereavement process [31,32,36], which is largely missing from the service descriptions [66]. Yet this is an important topic considering the increased mortality rate due to HIV/AIDS. The studies revealed that the social context influences the patterning of grief, the symptoms it generates and the emotions displayed. The data presented on mourning behaviour among the Zulu [32,48] showed that the expression of grief completely disappeared after the funeral and this raises doubts about the appropriateness of medicalised models of grief. The general literature has dealt with bereavement interventions mainly in terms of counselling. The studies also suggested alternative therapies based on communal reminiscence, where memories are retained instead of being discharged when expressed.

Cultural issues are central to people's illness concerns and healing practices and become especially important at the EoL. Providing a more comprehensive understanding of EoL care in different cultural contexts is an ethical enterprise especially in settings with limited resources and shortages of health personnel. It is necessary to subject the day-to-day healing/care practices to critical analysis in order to find suitable solutions for the challenges on the ground. Important questions to consider include: who is ill enough to receive palliative care or scarce medication? what are the limits of the responsibilities of carers? Daily practice in such different settings will inevitably complicate the remit and goals of palliative care itself and throw different light on moral notions as autonomy, or quality of life, so central to Western ethics.

### Strengths and limitations of the review

To the authors' knowledge, this is the first article to systematically review the qualitative literature on EoL care in sub-Saharan Africa. Furthermore, the search strategy used ensured the inclusion of studies employing qualitative research methods published in both biomedical and social science periodicals. Despite efforts being made to contact the authors of these studies, the full text versions of eleven studies could not be accessed. These studies included five articles on home based care in Zambia, Namibia, Kenya and Rwanda, three studies (from an unspecified country) on the experiences of healthcare staff, one on AIDS orphans in Kenya, one on the challenge for hospice care in Ghana, and one on palliative care guidelines for AIDS patients. However, the absence of these studies does not detract from the themes highlighted in the review or from the implications that the identified themes have for future research. The six studies, excluded because they were not published in English, based on the other exclusion criteria, would not have been included even if they had been in English. Although the exclusion of unpublished research may have biased the findings, it was necessary to ensure a minimum level of research quality. However, inevitably the breadth of topics addressed by the included studies limits the scope of the findings.

## Conclusion

This review contributes to the evidence-base on EoL care in sub-Saharan Africa by appraising the evidence from the qualitative literature on the experiences of those involved and the wider social and cultural context. The studies support and complement the findings from quantitative research [8].

Key findings from the qualitative synthesis were: to date, research has focused on HIV/AIDS and other illnesses are under-represented in the literature; care needs should be assessed from a household perspective; the assumption that, in African caring systems, the capacity of the extended family to withstand crisis is inexhaustible is baseless; actors, based in the community, home or the formal health care system, providing EoL care face a range of challenges yet some carers experienced benefits; home and community based care is important, but support from the formal health system is also required; place of care preference is complicated and more research is required; there is a paucity of research dealing with cultural issues at the EoL in African settings, yet these issues influence expectations and treatment decisions about EoL care (including pain management); stigma associated with illness at the EoL and carers is a topic that requires emphasis in future research; and, although there is a lack of research dealing with bereavement, it is apparent that social context influences the patterning of grief, the symptoms generated and the emotions displayed.

This review confirms that research, which is needed to inform adequate EoL care in Africa, is at an early stage and many research areas need to be explored. These areas give direction to relevant themes necessary to build a research agenda on socio-cultural issues at the EoL in sub-Saharan Africa. It is essential to generate this type of evidence alongside clinical and health services data, to ensure that EoL service provision is effective, appropriate, acceptable, in other words, ethical and culturally competent.

Although not yet fully documented, and not systematically evaluated, EoL care in sub-Saharan Africa is now finding its own approaches, and strategies depending on the conditions and constraints imposed by its geographical, economic, political and cultural context. These experiences are beginning to be acknowledged as a source to inform care in other parts of the world, including the West. Such experiences, gained as part of the unique conditions and challenges that EoL care practice faces in Africa, will provide an impulse to clarify and expand the defining features of EoL care. The insights gained from the evidence generated assist in broadening the discipline beyond traditional borders of more disease-specific knowledge and beyond familiar settings.

## Competing interests

The authors declare that they have no competing interests.

## Authors' contributions

MG and LS wrote the manuscript. MG, LS, CP and RP designed the search strategy. LS and CP undertook the search. All authors revised, commented on the manuscript and approved the final version

## Pre-publication history

The pre-publication history for this paper can be accessed here:

http://www.biomedcentral.com/1472-684X/10/6/prepub

## Supplementary Material

Additional file 1 **The search strategy**. Details of the search terms used and the journals in which hand-searches were carried out. Click here for file

Additional file 2 **Data extraction table**. A table containing the data extracted from each of the included articles and each article's grading score. Click here for file
