# Supplementary material for: End of life care in sub-Saharan Africa: a systematic review of the qualitative literature
Source: BMC Palliat Care. 2011 Mar 9;10:6. doi: 10.1186/1472-684X-10-6 (PMC3070681; doi:10.1186/1472-684X-10-6)
Supplement: Additional file 1 — The search strategy. Details of the search terms used and the journals in which hand-searches were carried out. [file 1472-684X-10-6-S1.DOC]

**Additional file 1. Search strategy**

The following search terms were used:

((Africa OR African OR Angola OR Benin OR Botswana OR Burkina Faso OR Burundi OR Cameroon OR Cape Verde OR Central African Republic OR Chad OR Comoros OR Congo OR Cote d'Ivoire OR Djibouti OR Eritrea OR Ethiopia OR Gabon OR Gambia OR Ghana OR Guinea OR Kenya OR Lesotho OR Liberia OR Madagascar OR Malawi OR Mali OR Mauritania OR Mauritius OR Mozambique OR Namibia OR Niger OR Nigeria OR Rwanda OR Sao Tome OR Principe OR Senegal OR Seychelles OR Sierra Leone OR Somalia OR South Africa OR Sudan OR Swaziland OR Tanzania OR Togo OR Uganda OR Zambia OR Zimbabwe) NOT (African-American OR African American) AND (palliative OR dying OR end of life OR end-of-life OR terminal* OR advanced diseases OR life threatening) AND (care OR caring OR care giver* OR carer OR hospice) AND

(qualitative OR social research OR ethnograph* OR anthropolog* OR narrative OR focus group* OR interview* OR perception*))

(The last search group was excluded from the IBSS search. In some databases the search had to be divided into two sections and merged owing the limited number of Boolean search terms permitted. Due to the limits of the search engine, the search undertaken in the database African Journals Online used the following terms: palliative OR dying OR end of life OR end-of-life OR terminal* OR advanced diseases OR life threatening.)

All-term abstract, key word, or topic searches (in this order of preference) were undertaken depending on the data base design.

Hand searches were conducted from 2004 to August 2010 in the following journals:

Tropical Medicine and International Health.

AIDS care,

Anthropology and Medicine,

BMC Palliative Care,

International Journal of Palliative Nursing,

Journal of Pain and Symptom Management,

Journal of Palliative Care,

Journal of Palliative Medicine,

Medical Anthropology Quarterly,

Palliative and Supportive Care,

Palliative Medicine,

Progress in Palliative Care,

Social Science and Medicine,
