# Supplementary material for: End of life care in sub-Saharan Africa: a systematic review of the qualitative literature
Source: BMC Palliat Care. 2011 Mar 9;10:6. doi: 10.1186/1472-684X-10-6 (PMC3070681; doi:10.1186/1472-684X-10-6)
Supplement: Additional file 2 — Data extraction table. A table containing the data extracted from each of the included articles and each article's grading score. [file 1472-684X-10-6-S2.DOC]

**Additional file 2. Data extraction table**

| **Study** | **Methods** | **Article quality** | **Study Aim** | **Participants** | **Participant type*** | **Location** | Main themes / concepts | **Conclusions *relevant to the care model in Africa*** |
| --- | --- | --- | --- | --- | --- | --- | --- | --- |
| Akintola 2010 | Qualitative interviews | 33 | To explore volunteer caregivers’ perceptions of rewards of providing care to people living with AIDS. | Volunteer care-givers (55) | CG | South Africa | Rewards associated with providing care. Choice and social exchange theory – emphasises interdependence in dydadic relationships and that people try to maximise their rewards from a relationship and mimise their losses. | Volunteer carers receive different kinds of rewards as part of their care-giving experiences. The rewards may recompense to a certain extent the burden that the carer experience. Rewards may act as motivation for carers to continue their involvement with care organisations with limited funds. The model of home-based care should have a dual focus – providing rewards for volunteers and enabling them to provide care. |
| Beck 2000 | In-depth interviews, focused interviews, site visits | 29 | To evaluate cultural and other factors influencing cancer pain management in South Africa. | Representatives from government, NGOs, higher education, pharmaceutical industry, statutory bodies, professional associations, health care providers (62) | HP | South Africa | Multiple dimensions of cancer pain. Cultural beliefs and practices. Factors influencing cancer pain management (standards, knowledge, resources, communication and the patient-provider relationship, teamwork and professional relationships). | Impact of cultural beliefs (of cause and pain of cancer). Cultural differences between HCP and patients can affect care. Importance of standards. Need for education about pain management. Need for resources. Organizational problems affect care. |
| Bester 2006 | In-depth interviews | 31 | To explore nurses’ experience of dying process of terminally ill AIDS patient and to formulate guidelines. | Nurses of terminally ill patients (10) | HP | South Africa | Factors that affect the pace of dying include: diagnosis, going home, nutrition, socio-economic status, specific signs and symptom. Medical treatment can be an issue. AIDS patients have specific physical and behavioural needs e.g. contact, wanting to go home. Nurses’ conduct can affect relationship between patient and his/her family and sometimes nurses do not know how to act towards family. Nurses feel powerlessness, emotional discomfort, sadness and fear. Value judgements such as women as victims, young as foolish, criticism of family, Christian empathy. | Nurse education about communications skills of life and death. Support for nurses. Further research of nursing skills. |
| Bor 1993 | Structured interviews using content analysis | 25 | Cross cultural study of Kaposi’s sarcoma (KS) to identify contextual and cultural differences, the main fears of patients and others, practical ways of addressing and coping with stigma, and methods of reducing distress before symptoms occur. | Patients, relatives and staff (20 Zambia and Botswana) *(11 USA) (8 UK)* | HP, CG, P | Zambia  Botswana  USA  UK | Setting and culture mediate social responses to KS Patients in Africa reported less stigma than those in the West, had far less fear of rejection from family and were less concerned about personal appearance. Issues of secrecy and effective treatment were relevant to both groups. Once treatment was no longer possible, there was a greater emphasis on counselling. | Counselling related to stigma, secrecy, treatment, cosmetic application, the effects of family and fears of disfigurement may be beneficial. |
| Brouwer et al. 2000 | Focus groups, in-depth interviews, review of counselling reports | 27 | To explore the problems, worries and needs of caretakers of HIV-infected children in Uganda and to explore ways to improve support. | Caretakers and counsellors (37) | CG | Uganda | Aspects of pre-test counselling, psychological stress, health of the child, material problems, future preparations. | Counselling at health centre should be provided as well as support and advice about HIV infection, proper childcare, and material problems. |
| Chimwaza and Watkins 2004 | Semi-structured interviews *(informed by survey and qualitative data from larger study)* | 34 | To investigate perspectives of caregivers of those with AIDS. | Caregivers of very ill people (15) | CG | Malawi | AIDS not formally diagnosed or named by caregivers but suspected. Caregivers tried to give best care possible and received social, moral, physical, and financial support from kin and community. Burden of care giving was seen as minimal because patients were close relatives and died relatively quickly. | Policy recommendations (though impractical considering current political situation): respite care for caretakers; training in basic hygiene and clinical aspects of AIDS for caretakers; home-based care for poor, rural patients in sub-Saharan Africa is the best and only option. |
| De Saxe Zermelo et al. 2006 | Focus groups, site visits, observation  *(survey)* | 28 | To understand the needs, fears and motivations of frontline care workers at a home-based care site. To explore attitudes to voluntary HIV counselling and testing, and emotional impacts of work. | Care workers (37) | HP | South Africa | The majority of care workers were sad or upset due to work often. The weekly care support meetings were very beneficial. Community stigma of HIV, fear of family reactions and fear of illness prevented many from taking HIV test. | Care workers are willing to raise orphans but funds need to be provided to build additional rooms. HBC organisations need to explore funding for transport of patients in critical conditions. Care workers need more support and information about accessing government grants. Care workers should have training in all aspects of their work. Expansion of care worker support groups should be explored. |
| Demmer 2006a | In-depth interviews | 36 | To give a voice to family caregivers of terminally ill AIDS patients. | Family caregivers (18) | CG | South Africa | Stress of caring made worse by: stigma and denial of HIV/AIDS; lack of formal and informal support | Greater emphasis on community-based interventions including training, interventions to reduce stigma, and encouragement of male participation in care giving. |
| Demmer 2006b | In-depth interviews | 29 | To examine how (professional) caregivers help clients deal with AIDS-related loss and grief and the personal impact it has. | Professional caregivers (8) | HP | South Africa | Client-centred issues (e.g. poverty, reluctance to share feelings, support groups) and Staff-related issues (e.g. stress, training, salaries). CG feelings can affect client care | Systematic research needed to explore worksite support of professional care-givers in South Africa. Need to develop viable ways to retain staff and determine best practice for helping individuals bereaved by AIDS (in South Africa). |
| Demmer 2007 | Semi-structured interviews | 32 | To explore the bereavement experiences of AIDS-related loss. | Bereaved individuals (due to a death from AIDS) (18) | C | South Africa | Survival needs overtook grief, economic impact of loss on household, burden of care for women, child welfare concerns | Further research needed of social, economic and political factors that influence AIDS-related bereavement in South Africa. Need development of interventions. |
| Dieleman 2007 | In-depth interviews, focus groups *(Self-administered questionnaires)* | 36 | To explore the perspectives of HIV/AIDS from the perspectives of health workers. | Health workers managers and volunteers (34 in-depth interviews) (5 focus groups) | HP | Zambia | HP feared infection, kept quiet if they were HIV positive, were emotionally exhausted, and lacked support from their managers. Managers also felt inadequate dealing with staff that were HIV positive and lacked guidelines or policies to help manage the situation. | HPs need better training and support. Specific workplace programmes could help address stigma in the workplace. Infection control measures need to be communicated and enforced to reduce fear of infection. Managers need guidelines and support. |
| Dilger 2008 | Case studies | 21 | To explore how moral perceptions of HIV/AIDS are related to disease interpretation, patient caring and burial in the context of migration. | HIV-infected men and women and their relatives | P | Tanzania | Care and burial of dying for someone with AIDS tied into local moral order of turning a bad illness into something positive. For example, becoming more religious, providing a good funeral, re-establishing kinships. Respondents cared less about reducing pain of body, and more about ‘moral practice’. | Care of someone dying from AIDS may be perceived in relation to rectifying perceived immoral cause of illness, and relates to community and kinship relations |
| Downing and Kawuma 2008 | Interviews, focus groups, observations, research diary, document analysis | 34 | To evaluate modular HIV/AIDS palliative care programme. | Participants from the programme | HP | Uganda | Positive impact at patient level, community level, participant level, district level. | Evaluation shows that programme is effective but content, logistics, how training is delivered and other training changes need to be made. |
| Downing 2008 | Case studies, interviews, focus groups, observations | 34 | To determine the effectiveness of the education strategy and explore what had taken place in order for any change in practice to be seen. | Trainers and participants | HP | Uganda | Change was brought about by: teamwork; the need to develop PC clinical skills and the issue of availability of essential drugs for PC; issues around stigma, blame and attitudes; the need to integrate PC services into already existing structures. | The ‘Nankya model’ needs to be validated in other settings for the development of PC in rural areas. |
| Ens et al. 2008 | Interviews | 33 | To describe the hospice care referral system in Western Cape Province from perspective of professionals using it. | Doctors (7), nurses (14) and social workers (8) | HP | South Africa | There was a lack of referral process, a lack of standardization and a lack of knowledge about referrals. | A more transparent referral system is needed. Hospices should make their goals clearer. Research needed about referral patterns in relation to changing nature of HIV/AIDS epidemic. |
| Fourie 2008 | Semi-structured interviews | 38 | To consider information needs and behaviours of patients with cancer in palliative care. | In and out patients, family members, oncologists, a doctor specialised in palliative care, nursing staff (25) | P, HP | South Africa | Patients and family members varied in the amount, type and expression of information they wanted but study was unable to deduce precise needs and behaviours. | Unexpressed needs need addressing and further research, including other disciplines, can contribute to understanding better information needs. |
| Grant et al. 2003 | Semi-structured interviews | 32 | To construct accounts of the needs of dying patients and their carers. | Patients (32) and are-givers (24) | P, CG | Kenya | Themes concern understanding, origin of, and reluctance to talk about illness, and physical (pain relief), emotional, social, spiritual and financial needs. | Spiritual and social support needs are met but pain relief, assistance in care and money are unmet needs. These needs need to be implemented in a culturally appropriate way to ensure a good death. |
| Harris et al. 2003 | Participant observation, semi-structured interviews, review of documents | 32 | To explore physicians’ decisions concerning the disclosure of diagnostic, prognostic, referral information that might potentially harm patients with cancer or their families and the effect of cultural, social, and economic factors on how this information is handled. | Health professionals (37) | HP | Tanzania | Expatriate and Tanzanian physicians practicing in North Tanzania often had different approaches to informing patients of their diagnoses and prognoses. | The use of the Tanzanian way of counselling the patient seems to be an acceptable and culturally appropriate approach to disclosure as long as this method brings patients to a sufficient level of understanding of their condition. |
| Hosegood et al. 2007 | Observation | 32 | To document the experiences of households directly affected by HIV and AIDS in a rural area. | Households (12) | CG | South Africa | Experiences highlighted: impact of multiple episodes of HIV related illnesses and deaths within (and outside) the household; stigma connected to HIV/AIDSI; impact of HIV/AIDS compounded by other illnesses and deaths. | Quantitative assessments of the impact of HIV/AIDS do not show complete household history of experiences of death and illness and could also be improved by considering illness episodes outside of the household. |
| Hunter 2005 | Ethnographic: repeated home visits, formal and informal interviews, household event mapping, participant observation, focus groups | 26 | To ascertain what provision of care and assistance ill people in need of care are receiving, and to compare this to government’s care policy. | Households (36), service providers, community members | CG, P, C | South Africa | Views reported on assistance from hospitals, clinics, disability grant, private provision, community caregivers and lived experience of providing care. | Policy not working – people are receiving no or little home support. Better policy should be a priority e.g. increasing role of community care givers. |
| Kang’ethe 2009a | Focus groups, interviews, participant observation | 26 | To discuss the challenges influencing the care provided as part of the community home-based care programme for persons living with HIV/AIDS and other terminal illnesses. | Primary care givers (82), supervising nurses (4) and community home-based care coordinator (1) | CG, HP | Botswana | Challenges faced by carers of people living with HIV/AIDS are detailed and discussed in relation to other southern African countries. | Carers are predominantly elderly female relatives who often lack the resources – adequate education, income, food, community support, shelter, and sanitation – to provide quality care to their patients. |
| Kang’ethe 2009b | Focus groups, interviews, participant observation | 26 | To evaluate the contribution of traditional healers in car-giving for persons living with HIV/AIDSTo assess the challenges involved managing HIV/AIDS using the allopathic and non-allopathic healing systems. To compare the allopathic and non-allopathic healing. | Primary care givers (82), supervising nurses (4) and a community home-based care coordinator (1) | CG, HP | Botswana | General discussion of “positive” and “negative” aspects of traditional healers (not based on the collected data). Use of traditional healers by the patients that the carers treat discussed. | Use of traditional healers for AIDS care is becoming less popular. This is possibly due to advocacy on the part of biomedicine against the used of traditional medicine. |
| Kang’ethe 2010 | Focus groups, interviews, participant observation | 25 | To explore the attitudes and perception of family and community palliative care givers pertaining to volunteerism. | Primary care givers (82), supervising nurses (4) and a community home-based care coordinator (1) | CG, HP | Botswana | Who are the carers of people living with HIV/AIDS and why do they carry out this work. | Carers are motivated by kinship ties, patriotism associated with government calls to the public to bear the burden of care-giving, religious ideals, and local ideals of volunteerism linked to Setswana culture. |
| Kell and Walley 2009 | Semi-structured interviews | 33 | To understand the perception of nurses of palliative care in Lesotho in the era of ARV availability. To discuss the need for palliative care, through analysis of the components of the WHO definition. To explore whether the nurses think that the WHO IMAI guidelines are a useful tool for the implementation and scaling up of palliative care services. | Nurses (10) and key informants from the ministry of health and the Christian Health Authority of Lesotho (6) | HP | Lesotho | Nurses’ knowledge of palliative care depended upon the training they had received. However, the need to holistic care was recognised for HIV-positive patients even in the presence of ARVs, though unclear whether such care was provided. Opinions on the quality of pain management for HIV-positive patients varied. Palliative care was a little-known element of the IMAI. | Palliative care remains necessary in the era of ARV availability. However, it is significantly lacking in Lesotho and it will remain so without political will from the ministry of health. The WHO IMAI is a useful tool for scaling-up HIV care and palliative care provision, however it is not being adequately used and a great focus on a comprehensive care package is required. |
| Lindsey et al. 2003 | Interviews | 36 | To investigate the experiences and impact on young girls and older women caring for family members living with HIV/AIDS and other chronic and terminal illnesses. | Family caregivers (35), community home-based care team members and government officials (35) | CG, HP | Botswana | Older women felt overwhelmed, exhausted, malnourished, depressed and neglectful of own health. Young girls missed school, were abused, sexually exploited and depressed. Caregivers experienced poverty, social isolation, stigma, psychological distress and lack of basic care giving education. | Specific recommendations made to improve education, services and care giving. |
| Matukala-Nkosi et al. 2006 | Focus groups  *(Surveys)* | 29 | To examine factors affecting caregivers of PLWA. | Female Caregivers (12), HBC workers (6) and key informants (5) | CG, HP | Democratic Republic of Congo | Become a caregiver because of love and sense of responsibility, traumatic and stressful experiences of being a CG, lack of support, CG health, opinions of HP and community leaders re care giving. | Family CG should be integrated into HIV/AIDS programmes and budgets. This CG population should be targeted as a priority in local health and social services. Funds (i.e. from national and international agencies) should be directed to female CG. Thinking needs to shift to the family rather than the individual. |
| Moore and Henry 2005 | Interviews | 34 | To examine the experiences of older caregivers to people living with IV/AIDS. | Informal caregivers over 50 years old (50) | CG | Togo | Caregivers’ feelings of personal inadequacy, community stigma to person with HIV/AIDS, financial strain, and adaptations in intimate behaviour. Old age compounded difficulties and most wanted an institutional solution to relieve burden of care. | Policymakers need to incorporate caregivers into their policies and services. |
| Mtalene and Preston-Whyte 1993 | In-depth interviews | 21 | To examine what the experiences are of terminal illness among Zulu speaking patients, their families and their caregivers. | Hospital patients (10) | P | South Africa | History of illness and treatment, patient and family reactions to diagnosis, relationship with caregiver, family support, role of religion and dreams, reactions and rituals of family upon death of patient. | Caregivers need to discuss dying with patient though caregivers lack skills and recognition of cultural beliefs. Specialist nurse role should be created. Improve training of care for dying patients and cultural sensitivity. |
| Muntree and Maharaj 2010 | Focus groups (Household survey) | 34 | To provide insights into the multiple impacts of the HIV/AIDS epidemic on the lives of older men and women. | Older community members (8 FGDs of 6-8 participants) *(974 community members)* | P | South Africa | The roles of older persons in KwaZulu-Natal in caring for family members infected with and/or affected by HIV/AIDS and their perceptions of these roles. | Older persons are required to take over a range of roles as care-givers, taking care of family members infected with and affected by HIV. The strenuous nature of these roles may also influence their own health. They are aware of the mode of transmission of HIV but, due to a lack of resources, are unable to protect themselves whilst they care for HIV-positive family members. |
| Murray et al. 2003 | Interviews  *((Longitudinal study (Scotland) Cross sectional study (Kenya))* | 31 | To describe the experiences of illness needs and use of services of patients with incurable cancer. | Patients with advanced cancer and their main informal carer (24) | CG, P | Kenya  *Scotland (20)* | Comparison between Kenya and Scotland. For Kenyan patients, experience dominated by pain. Needs, attitudes and priorities differed. | Despite physical needs being unmet, many psychosocial needs were met in Kenya. Physical needs need to be met without destroying capacity of family etc. to meet other needs. |
| Ndaba-Mbata and Seloilwe 2000 | In-depth interviews | 31 | To assess the level of knowledge available to families giving home care to terminally ill relatives. | Families giving care (15) | CG | Botswana | Caregivers’ perception of care, caregiver level of knowledge, rights of ill persons vs. rights of caregivers, management of symptoms. Families lacked knowledge and skills for providing appropriate care, were not aware of available resources and lacked professional and material support. | Families should have guidance and support. A good referral and follow-up system for home-based care should be implemented with monitoring and evaluation. |
| Niehaus 2007 | Open ended interviews, observations | 28 | To explore social and cultural factors that have undermined effective care and treatment for PLWA. | Community members (25) | C | South Africa | Public health messages have focused on preventing AIDS rather than treatment, increasing perception that AIDS is incurable and fatal. Alongside this, the church constructs AIDS as a new kind of leprosy (but without cure). Stigma of AIDS as due to sexual immorality unlikely because promiscuous sex or multiple partners is not taboo, people talk freely about sex and other STDs. Instead AIDS-related stigma is linked to its terminal nature and terminal illness represents a social death. AIDS is also visually like a decomposing body. | AIDS needs to be redefined as a manageable chronic disease, and public health and schools need to stress not just prevention but also that AIDS is manageable. |
| Nnko 2000 | In-depth interviews | 23 | To examine experiences of 21 carers of multiple chronically ill people (with AIDS). To assess whether the quality of care increased with subsequent patients, due to previous experience in the absence of professional support. | Caregivers (21) | CG | Tanzania | Caregivers talked about: the burden of care, including not knowing about HIV status early enough in patient’s illness; community based support systems; treatment seeking; advice to other care givers; what they had learnt from their experiences. | Burden of care is mainly born by women. Outside support may depend on relationship patient had with others before becoming ill. Economic support needed. Use of traditional healers declines with care giving experience. Carers would benefit from knowing HIV status as early as possible. No counselling was received about how to care; this would be useful. |
| Notter et al. 2007 | Interviews | 27 | To evaluate programme of HBC workshops with female village elders. | Nurses (17), Clinical medical officers (9) and female elders taking part in the programme (17) | HP, CG | Kenya | Families and HP accepted women’s role and terminal care was improved. CBDs (community based distributors) crossed boundary between HP focus on medical care and local, holistic needs and supported the programme. |  |
| Nzioka 2000 | In-depth interviews | 26 | To examine how people make sense of death from HIV/AIDS. | People with HIV (14), clergy members and lay people | P | Kenya | AIDS seen as a shameful death and spiritually permanent which leads to denial, concealment, and lack of financial and social support at funerals. | Need to de-stigmatise HIV/AIDS through education and community based care. |
| Olenja 1999 | Focus groups, key informant interviews, social mapping, written compositions by school children *(Survey)* | 25 | To assess community attitudes towards HIV/AIDS and home based care. | Community members (6 focus groups), community leaders (3 focus groups), village health committees (3 focus groups), people living with AIDS (3 focus groups), health personal (6 interviews) and head teachers (6 interviews) | CG, C, P, HP | Kenya | Institutional care preferred to HBC due to lack of knowledge. Fear due to lack of knowledge increases stigma of people with AIDS. Families were not caring for relatives because they were the best carers, but instead because they were ‘stuck’ with patient and had no other support. | Community work needs to be done to lessen burden. More training at family and community level needed. |
| Posel et al. 2007 | Focus groups | 30 | To examine how a rural community are impacted by, and comprehend high rates of AIDS-related death. | Community members (12 groups of 6-8 participants) | C | South Africa | Cause of deaths rarely referred to as AIDS. Instead, these ‘bad deaths’ are caused by erosion of cultural norms and traditions. Physical illnesses demonstrate a social/cultural illness and related to cultural change (e.g. democracy). | Information can be recited without being internalised into beliefs about choices and actions and therefore has implications for public health messages. Some fear around western medicine. |
| Roby and Eddleman 2007 | Focus groups, interviews  *(Questionnaire)* | 30 | To explore the plans and options available to Mozambican mothers in regard to the care of their children after death. | Mothers with terminal illnesses (102), community activists (9), local research assistants (3), government leaders and directors of NGO-sponsored orphanages (2) | P | Mozambique | Most assumed extended family would care for children but none had a clear plan. Women hoped for government assistance for children after death and preferred orphanages over other options though orphanages are full and there is very limited government assistance. | Collaboration needed between community, national and international bodies to meet needs of orphans. |
| Selman et al. 2009 | Semi-structured, qualitative interviews | 37 | To explore the information needs of patients with progressive, life-limiting diseases and their care-givers in South African and Uganda. To inform clinical practice and policy in this emerging field. | Patients (90) and family care-givers (38) (from 4 palliative care services in South Africa and 1 in Uganda) | P, CG | South Africa,  Uganda | Communication and information, a general theme in the qualitative data was broken down into the following sub-themes: sources of information; information need; impact of unmet information needs; communication with general healthcare staff; barriers to effective provision of information. | Patients and care-givers often lacked adequate information. This adversely affected their ability to cope with their situation, directly affecting care-givers’ ability to care for patients and patients’ ability to care for themselves and plan for the future. This was also a source of anxiety. Information provided should be tailored to the individual, offered in a proactive, reflexive, open and honest way. Information provision should be recorded and should be available for both patient and care-giver. |
| Smit 2005 | In-depth interviews | 37 | To report on the perceptions and experiences of nurses caring for people living with HIV/AIDS in South Africa. | Nurses (35) | HP | South Africa | Negative perceptions and experiences were: helplessness, emotional stress and fatigue, fear, anger and frustration, occupational-related concerns. Positive perceptions and experiences were: empathy, and self-fulfilment. | Nurses need more specific training and updates regarding care of people with HIV/AIDS, and more occupational support. |
| Ssengonzi 2007 | Focus groups, in-depth interviews | 35 | To describe challenges faced by elderly caregivers of relatives with HIV, and their uninfected children. | Elderly members of the community (160-200 focus group participants), elderly men (11 interviews), elderly women (16 interviews) currently or formally caring for a child with HIV/AIDS | CG | Uganda | Older caregivers faced economic burden of reduced savings for cost of care of both ill person and orphaned children, has health problems (especially women), were worried about becoming infected themselves, lacked skills, and were not optimistic about the future. | Interventions need to provide basic needs such as gloves, training in care giving practices, and counselling skills for dealing with depressed patients and bereaved orphans. |
| Ssengonzi 2008 | In-depth interviews | 27 | To examine the changes in household structure and living arrangements of older persons who care for people who live with HIV/AIDS, orphans and vulnerable children affected by HIV/AIDS. To discus the implications on older persons’ well-being. | Older persons (27) (men and women) | CG | Uganda | Living arrangements and well being of elderly people affected by the care they give to people infected with and children affect by HIV/AIDS. Living arrangements can change drastically – changes that are often dependent on the gender, age and marital status of the person living with HIV/AIDS. Care-giving is not stigmatised, however fears of HIV disclosure prevent patients from accessing appropriate care. | The role of elder persons in caring for persons living with HIV/AIDS will remain critical for the foreseeable future and understanding their needs and how to improve their well-being are important research topics. |
| Thomas 2006 | Solicited diaries | 33 | How duty of caring impacts on upon physical and psychological well-being of ill people and their carers. | Ill people (7) and their care givers (7) | CG, P | Namibia | Dependence while ill is a key factor influencing patient treatment, identity and well-being. | Appropriate local interventions need to be identified to reduce burden of care. Need to challenge notion that duty of care rests with women. |
| Uwimana and Struthers 2008a | Interviews, focus groups  *(Questionnaires)* | 28 | To identify palliative care needs of PLWHA. | Co-ordinators of HIV/AIDS unit (4), PLWHA (12) *(Questionnaire: PLWHA, Healthcare workers and co-ordinators of HIV/AIDS units (300))* | P, HP | Rwanda | Biggest unmet needs were financial assistance, home based care, nutrition and pain relief. Psychological support, even when provided, is not seen as very helpful. | PC programmes need to be revised, especially pain control, financial needs and psychological support. |
| Uwimana and Struthers 2008b | Interviews, focus groups  *(Questionnaires)* | 28 | To investigate where HIV/AIDS patients want to be cared for in the terminal phase of illness. | Co-ordinators of HIV/AIDS unit (4), PLWHA (12) *(Questionnaire: PLWHA, Healthcare workers and co-ordinators of HIV/AIDS units (300))* | P, HP | Rwanda | Of participants, 67% indicated that they would prefer to be looked after in hospital during the terminal phase of illness and 26% indicated that they would prefer to be looked after at home. Only 2% of participants indicated they would prefer to be looked after at a home-based care centre and less than 1% of participants preferred to be looked after at a church. | Home-based care programmes need to be able and prepared to meet all the needs of HIV/AIDS patients. |
| Uys 2003 | Semi-structured interviews, observations  *(Questionnaires)* | 32 | To explore the realization of counselling, palliative care and terminal care of PLWHA. | Patients, carers, health professionals and community members | P, HP, CG, C | South Africa | Need for longer-term counselling, largest amount of ‘good deaths’, were deaths at home, pain relief and care managed badly at hospitals and lack of trained PC nurses, nurses comfortable providing spiritual care to patients. | Counselling of AIDS needs to be developed. HBC improves dying experience for patient (though some patients are hospitalized to get food). Greater outreach programmes are required. |
| Uys 2002 | On site visits, interviews, collection of hospital statistics | 23 | To describe the practice of community caregivers in a home-based AIDS care project. | Community caregivers (16) | CG | South Africa | Community caregivers perceive that they are getting results but have problems with patients with psychiatric needs, and feel that their role and status is not acknowledged by the formal health services. | Outcome of HBC, and impact of counselling should be studied. |
| Van de Geest 2002 | Ethnographic fieldwork | 30 | To explore the views of older people about death. | Older people in the community | C | Ghana | Older people generally look forward to death. Traditional ideas of ancestorhood, reincarnation and modern Christian beliefs about life after death had little influence on their resignation. | Older people’s view of death is part of their status as elder in the community. |
| Van de Geest 2004 | Ethnographic fieldwork | 30 | To consider good and bad death in Kwahu-Tafo, Ghana. | Mixed, including community members | CG, C | Ghana | Outline intricacies of a good death and a bad death and ambiguities. Good death is essentially dying in peace. Five aspects to dying in peace: peace with other people; peace with own death; a ‘natural’ death; dying in a good place (e.g. home); the living are at peace with the death. | Good death has a social, psychological/spiritual, time and spatial aspect. Unlike Western (Dutch) culture, palliative care does not feature in a good end of life. |
| Waterman et al., 2007 | Participatory Action Research, focus groups | 35 | To evaluate HBC in Nyanza province, Keyna (this paper focuses on HP role in reducing stigma of HIV). | HBC professionals (50) | HP | Kenya | Power broking and mobilization, stigma as a social construction, community and structural interventions, educating and training people, historical context. | Stigma was a large barrier to HBC. Parker and Aggleton’s framework of stigma did fit with findings though it neglected the role of individual factors such as education and psychological distress. HP mostly challenged stigma at individual and community level. |
| Withell 2000 | Semi-structured interviews | 36 | To investigate the experiences of women living with HIV/AIDS. | HIV positive women (7) | P | Uganda | Loss and adversity (bereavements, multiple psychosocial losses, physical suffering), constructive living (living activities, rebuilding lives), future uncertainties (child care, HIV status of children). | Research around children issues (e.g. testing, preparing them for bereavement, custody) recommended. |

**NB: Where a study used mixed methods, this table only includes the qualitative findings.**

*CG – Caregiver (informal)

HP – Health professional

P – Person requiring/receiving palliative care

C – Community member

ICHC - Integrated community-based home care

HBC – Home-based care

CHBC – Community home-based care

PLWHA – Person living with HIV/AIDS
